# Systematic reviews and meta-analyses comparing mortality in restrictive and liberal haemoglobin thresholds for red cell transfusion: an overview of systematic reviews

**DOI:** 10.1186/s12916-020-01614-w

**Published:** 2020-06-24

**Authors:** Kevin M. Trentino, Shannon L. Farmer, Michael F. Leahy, Frank M. Sanfilippo, James P. Isbister, Rhonda Mayberry, Axel Hofmann, Aryeh Shander, Craig French, Kevin Murray

**Affiliations:** 1 School of Population and Global Health, The University of Western Australia, Perth, Australia; 2 Data and Digital Innovation, East Metropolitan Health Service, Perth, Australia; 3 Medical School and Division of Surgery, The University of Western Australia, Perth, Australia; 4 School of Health Sciences and Graduate Studies, Curtin University, Bentley, Australia; 5 Department of Haematology, PathWest Laboratory Medicine, Royal Perth Hospital, Perth, Australia; 6 Medical School, The University of Western Australia, Perth, Australia; 7 School of Medicine, The University of Sydney, Sydney, Australia; 8 Library and Information Service, South Metropolitan Health Service, Murdoch, Australia; 9 Institute of Anesthesiology, University Hospital Zurich, Zurich, Switzerland; 10 Department of Anesthesiology, Englewood Hospital and Medical Center, TeamHealth Research Institute New Jersey, Englewood, USA; 11 Australian and New Zealand Intensive Care Research Centre, Department of Epidemiology and Preventive Medicine, Monash University, Melbourne, Australia

**Keywords:** Anaemia, Red cell transfusion, Systematic review, Overview

## Abstract

**Background:**

There are no overviews of systematic reviews investigating haemoglobin thresholds for transfusion. This is important as the literature on transfusion thresholds has grown considerably in recent years. Our aim was to synthesise evidence from systematic reviews and meta-analyses of the effects of restrictive and liberal transfusion strategies on mortality.

**Methods:**

This was a systematic review of systematic reviews (overview). We searched MEDLINE, Embase, Web of Science Core Collection, PubMed, Google Scholar, and the Joanna Briggs Institute EBP Database, from 2008 to 2018. We included systematic reviews and meta-analyses of randomised controlled trials comparing mortality in patients assigned to red cell transfusion strategies based on haemoglobin thresholds. Two independent reviewers extracted data and assessed methodological quality. We assessed the methodological quality of included reviews using AMSTAR 2 and the quality of evidence pooled using an algorithm to assign GRADE levels.

**Results:**

We included 19 systematic reviews reporting 33 meta-analyses of mortality outcomes from 53 unique randomised controlled trials. Of the 33 meta-analyses, one was graded as high quality, 15 were moderate, and 17 were low. Of the meta-analyses presenting high- to moderate-quality evidence, 12 (75.0%) reported no statistically significant difference in mortality between restrictive and liberal transfusion groups and four (25.0%) reported significantly lower mortality for patients assigned to a restrictive transfusion strategy. We found few systematic reviews addressed clinical differences between included studies: variation was observed in haemoglobin threshold concentrations, the absolute between group difference in haemoglobin threshold concentration, time to randomisation (resulting in transfusions administered prior to randomisation), and transfusion dosing regimens.

**Conclusions:**

Meta-analyses graded as high to moderate quality indicate that in most patient populations no difference in mortality exists between patients assigned to a restrictive or liberal transfusion strategy.

**Trial registration:**

PROSPERO CRD42019120503

## Background

In 1999, the Transfusion Requirements in Critical Care trial [1] was published. This trial randomised critical care patients to either a restrictive or liberal red blood cell transfusion strategy. Patients assigned to the restrictive strategy were transfused if their haemoglobin concentration dropped below 70 g/L and concentrations were maintained at 70 to 90 g/L. Patients assigned to the liberal strategy were transfused if their haemoglobin concentration dropped below 100 g/L with concentrations maintained at 100 to 120 g/L. The authors concluded “a restrictive strategy of red cell transfusion was at least as effective and possibly superior to a liberal haemoglobin strategy”. Since that study, many randomised controlled trials (RCTs) evaluating restrictive and liberal red cell transfusion strategies in a variety of patient populations have been published. Subsequently, systematic reviews and meta-analyses synthesising the results of these trials were conducted.

Systematic reviews of RCTs are considered the highest level of evidence [2] and impact practice, the development of clinical guidelines, and policy making. An overview of systematic reviews collates and appraises the quality of previously conducted systematic reviews and meta-analyses. No overview is available for the impact of restrictive and liberal transfusion strategies. We conducted this overview to critically appraise and summarise the systematic reviews and meta-analyses describing red cell transfusion strategies published to date [3].

A potential misconception surrounding studies investigating pre-transfusion haemoglobin thresholds is that the intervention of interest is red cell transfusion. However, these studies are not designed to test the efficacy of transfusion as they do not compare red cell transfusion to placebo or another intervention. Rather, the intervention studied is the haemoglobin threshold, in other words whether and to what extent lowering the haemoglobin threshold for transfusion can be tolerated safely. Therefore, where studies demonstrate restrictive transfusion strategies are safe, the benefits include reducing the number of transfusions, reducing patient exposure to the hazards of transfusion, preservation of a finite resource, and reducing the significant hospital costs associated with transfusion [4].

Our objective was to compare and contrast evidence from systematic reviews and meta-analyses of the effects of restrictive and liberal haemoglobin threshold strategies on mortality. Specifically, our aim was to answer whether mortality differed between systematic reviews and meta-analyses of RCTs comparing restrictive to liberal haemoglobin thresholds for red blood cell transfusion.

## Methods

Overviews of systematic reviews are a relatively new area of research and a number of methodological approaches exist. Our protocol is consistent with expert recommendations published in a series of articles describing the development and evaluation of overview methods [5, 6]. The protocol was registered on PROSPERO (CRD42019120503) prior to commencing our review, underwent peer-review, and was published [7]. It includes the review question, literature search strategy, inclusion and exclusion criteria, and methods for assessing the quality of included reviews and the quality of evidence presented. Formal ethics approval was not required for this overview as we only analysed published literature.

### Data sources and searches

We searched MEDLINE, Embase, Web of Science Core Collection, PubMed (for prepublication, in process and non-MEDLINE records), and the Joanna Briggs Institute EBP Database on the 30 January 2019. A medical librarian (RM) developed our search strategy, and this process underwent internal peer review. In addition, we searched Google Scholar and contacted experts in transfusion literature to identify additional studies. Our literature search was restricted to studies published between 2008 and 2018. Details of our search strategy can be found in the Additional file 1.

### Study selection

#### Types of reviews

We included systematic reviews and meta-analyses of RCTs published in the English language between 2008 and 2018. We restricted our search to this period as we wanted to assess the most recent literature, and meta-analyses are frequently updated. We excluded abstracts, systematic reviews without meta-analyses, and systematic reviews and meta-analyses of observational studies. We also excluded earlier meta-analyses that had been subsequently updated within our time period. Reviews of observational studies were excluded because RCTs provide a complete summary of the effect different haemoglobin thresholds for red cell transfusion have on outcomes.

#### Participants

We included meta-analyses pooling patients randomised to red cell transfusion strategies, with the exception of meta-analyses of trials exclusively in neonatal and preterm infant populations.

#### Interventions/comparisons

We included meta-analyses of trials randomising patients to restrictive and liberal haemoglobin strategies as defined by the study authors.

#### Outcomes

The primary outcome of interest for this overview is mortality. We included any mortality time points reported within the included reviews, including reviews pooling mixed mortality time points. We also report red cell utilisation outcomes including the proportion of patients receiving a red cell transfusion and the mean number of units transfused. We a priori determined not to report morbidity outcomes. While important, the definition, grade, and severity of morbidity events pooled by systematic reviews and meta-analyses vary considerably, and as a result, interpretation is more subjective [8–10].

### Data extraction and quality assessment

We designed electronic forms to collect the key characteristics of included reviews. Two authors (KT, SF) independently screened titles and abstracts for inclusion. Discrepancies in article selections were resolved by discussion. Systematic reviews and meta-analyses are frequently updated as new trials are published. Where a review was updated, the most recent publication was included.

The data collection process was performed with two authors (KT, SF) independently extracting data from systematic reviews and meta-analyses and entering these on pre prepared data extraction forms. All disagreements in data extraction were resolved by discussion. When required, study authors were contacted for additional data or clarifications.

We used an electronic data extraction form to record data on author details, year of publication, clinical setting, inclusion criteria, exclusion criteria, number of participants randomised, number of included studies, mean units transfused, proportion of patients transfused, mortality time points reported, subgroups reported, description of individual trial interventions (haemoglobin thresholds) pooled, description of timing of interventions, and description of risk of bias assessments.

We assessed the methodological quality of included reviews in duplicate using the 16 domains described in the Assessing the Methodological Quality of Systematic Reviews (AMSTAR 2) tool [11]. This tool assesses important aspects of systematic reviews including addressing heterogeneity and investigating publication bias (Additional file 1: Table S1). Differences were resolved by discussion, and a full summary of results is presented in graphical format. We included information on whether individual systematic reviews and meta-analyses included a risk of bias assessment of individual trials, and what tools were used.

Two authors (KT and SF) independently evaluated the quality of evidence pooled within the systematic reviews and meta-analyses using a previously applied algorithm specifically developed to assign GRADE levels of evidence for overviews [12]. This algorithm uses the number of participants pooled, risk of bias, statistical heterogeneity, and the methodological quality of systematic reviews [13], to assess imprecision, risk of bias (trial quality and review quality), and inconsistency. Results are categorised into four levels of quality: high, moderate, low, or very low. More detailed information on how the final GRADE level is determined is presented in Additional file 1: Table S2.

We chose this approach to improve the transparency and consistency of our assessments [6]. Disagreements were minor in nature and resolved through discussion. Based on the definitions of quality of evidence grades presented in the GRADE Handbook (Additional file 1: Table S3) [14], we interpreted high and moderate GRADE levels to represent strong evidence. This approach has been previously described [15]. A low GRADE means that confidence in the result is limited as the true effect may be substantially different from the estimate.

### Data synthesis and analysis

We expected to find significant overlap of RCTs included in the meta-analyses. As we did not exclude any systematic reviews based on overlap, we decided a priori not to combine the results from the included meta-analyses and instead present the results descriptively in the text, figures, and tables. The results of included meta-analyses were presented as rate ratios with 95% confidence intervals. As per our protocol, we planned to convert any meta-analysis results presenting odds ratios to rate ratios. However, our primary outcome was mortality, and mortality events were low. Given the rate ratio, risk ratio, and odds ratio are approximately the same when comparing groups with low event rates, we made a decision not to convert odds ratios to risk ratios. Instead, we included the results of these relative risks as presented by the meta-analyses making a note of what measure was used.

Our analysis presents results comparing restrictive to liberal thresholds; therefore, the ratios and 95% confidence intervals of any meta-analyses comparing liberal to restrictive are inversed.

### Patient and public involvement

It was not possible or appropriate to involve patients or the public in this work as it involved a summary of research already conducted.

## Results

**Fig. 1:**
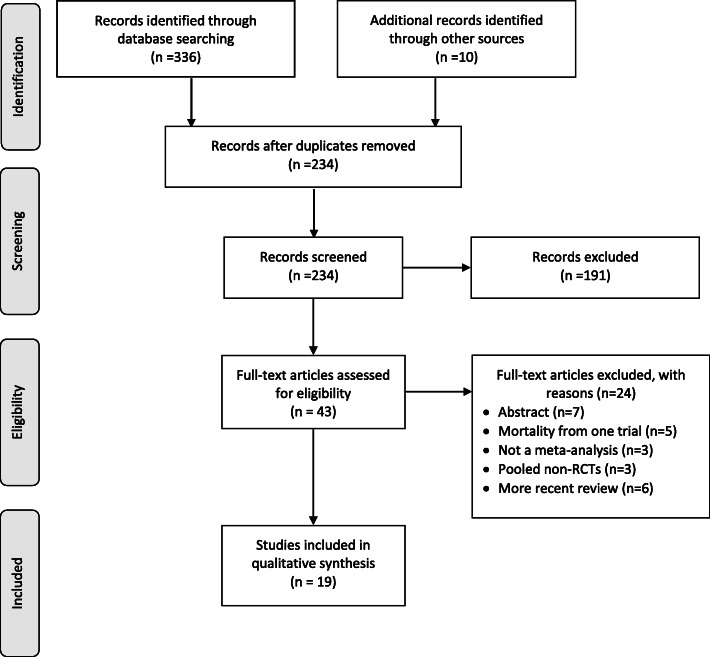
PRISMA flow diagram

Figure 1 summarises the number of studies identified, screened, and included in our study. Our database search identified 336 records, and a further 10 were identified from Google Scholar, with no additional studies identified after contacting experts in transfusion literature. After duplicates were removed, our literature search returned 234 records, of which 191 were excluded on abstract review. Forty-three full-text articles were retrieved and assessed for eligibility.

Of these, we excluded 24 studies [9, 16–38] for the following reasons: seven were abstracts [16, 21, 22, 24, 25, 27, 31], five presented mortality from one trial [28, 29, 32, 33, 38], three did not include a meta-analysis [19, 26, 34], three pooled non-randomised trials [20, 35, 37], and six were updated by more recent reviews (Additional file 1: Table S4) [9, 17, 18, 23, 30, 36]. Nineteen systematic reviews provided 33 meta-analyses that satisfied the eligibility criteria of our overview [8, 39–56].

Table 1 presents the characteristics of the included systematic reviews and meta-analyses. The number of individual trials pooled in each systematic review ranged from 3 [54] to 37 [40], with the total number of patients randomised ranging from 733 [47] to 19,049 [40]. The majority of systematic reviews pooled results from mixed medical and surgical settings (six reviews) [8, 40, 42, 43, 54, 56]. Following this, the most common clinical settings pooled were orthopaedic surgery (five reviews) [39, 44, 48–50], surgical and critical care (three reviews) [41, 45, 52], cardiac surgery (two reviews) [46, 55], acute upper gastrointestinal bleeding (one review) [51], critical care and acute coronary syndrome (one review) [53], and haematology/oncology (one review) [53].

**Table 1:** Characteristics of included reviews

First author	Year	Population	Total no. of patients	Total no. of trials	Proportion transfused RBCs	Mean difference in units transfused	Describes timing of intervention	Mortality time points pooled in meta-analyses	Subgroups presented for mortality outcome
Brunskill	2015	Patients undergoing surgery for hip fracture	2722	6	R: 38%, L: 96%*	NR	Y	30-day; 60-day; 90-day	
Carson	2018	Adults or children admitted for surgical or medical care	19,049	37	R: 50%, L: 81%	NR	N	30-day	Total; cardiac surgery; acute myocardial infarction
Chong	2018	Adult surgical or critically ill patients	10,797	27	NR	Critical care 1.7, surgical 1.3	N	30-day; late mortality (60 to 365 days); in-study	Critical care; surgical
Cortes-Puch	2018	Patients with and without cardiovascular disease	14,397	17	NR	NR	N	30-day or in-hospital	Cardiovascular disease patients hospitalised for non-cardiac reasons; for percutaneous coronary interventions; for cardiac surgery
Docherty	2016	Adults with cardiovascular disease not undergoing cardiac surgery	3033	11	R: 41%, L: 96%*	NR	N	30-day	Total; Stratified by cardiovascular disease
Gu	2018	Adult patients undergoing orthopaedic surgery	3968	10	NR	NR	N	30-day	
Holst	2015	Adults or children admitted for surgical or medical care	9813	31	R: 45%, L: 86%	1.43	N	Mixed (unspecified)	Low risk of bias trials
Hovaguimian	2016	Adult surgical or critically ill patients	12,052	31	NR	NR	N	Within 30 days	Cardiovascular disease undergoing cardiac or vascular procedures; patients with cardiovascular disease undergoing orthopaedic surgery; surgical and medical patients admitted to an acute care facility
Kheiri	2018	Patients undergoing cardiac surgery	9005	9	R: 52%, L: 76%	NR	Y	Within 30 days	
Luo	2018	Haematology/oncology	733	5	NR	0.33	N	60-day	
Mao	2017	Adults undergoing hip or knee surgery	3788	10	R: 36%, L: 74%	NR	N	30-day	
Melchor	2016	Critically ill adults admitted to intensive care units and/or with acute coronary syndrome	2159	6	R: 63%, L: 99%	NR	Y	Mixed (longest term)	Critical care; coronary artery disease
Mitchell	2017	Adults undergoing hip or knee surgery or hip fracture repair	3783	9	R: 39%, L: 81%	0.95	N	Mixed (unspecified)	
Muller	2018	Patients undergoing major orthopaedic surgery	3693	8	R: 36%, L: 82%	NR	N	30-day	
Odutayo	2017	Patients 16 years and older with acute upper gastrointestinal bleeding	1965	5	NR	1.73	N	Mixed	
Patel	2015	Adult patients undergoing cardiac surgery, adult and paediatric critically ill or noncardiac surgical patients	11,713	25	NR	NR	N	30-day	Cardiac surgery; non-cardiac surgery
Salpeter	2014	Adults or children admitted for surgical or medical care	2364	3	NR	1.98	N	In-hospital, 30-day, total	
Shehata	2018	Adults or paediatric patients undergoing cardiac surgery	9092	13	R: 53%, L: 78%	0.90	Y	Within 30 days	Adult patients; paediatric patients
Simon	2017	Adults admitted for surgical or medical care where a substantial proportion of the trial population was older than 65 years	5780	9	R: 52%, L: 77%*	NR	N	30-day; 90-day	

*R* restrictive strategy, *L* liberal strategy, *RBC* red blood cell, *NR* not reported

*Calculated from data presented in tables and figures

Of the 19 included systematic reviews and meta-analyses, 16 did not restrict their literature searches by language [8, 39, 40, 42–47, 50–56]. One review restricted their search to publications in the English language [49], while the language restrictions of the search strategies of two reviews were unclear [41, 48]. Eight reviews did not restrict their search strategy by publication status [8, 39, 40, 43, 44, 48, 54, 55], two reviews restricted their search results to trials published as full reports [45, 53], while the publication status restriction of the other nine reviews were unclear [41, 42, 46, 47, 49–52, 56].

**Fig. 2:**
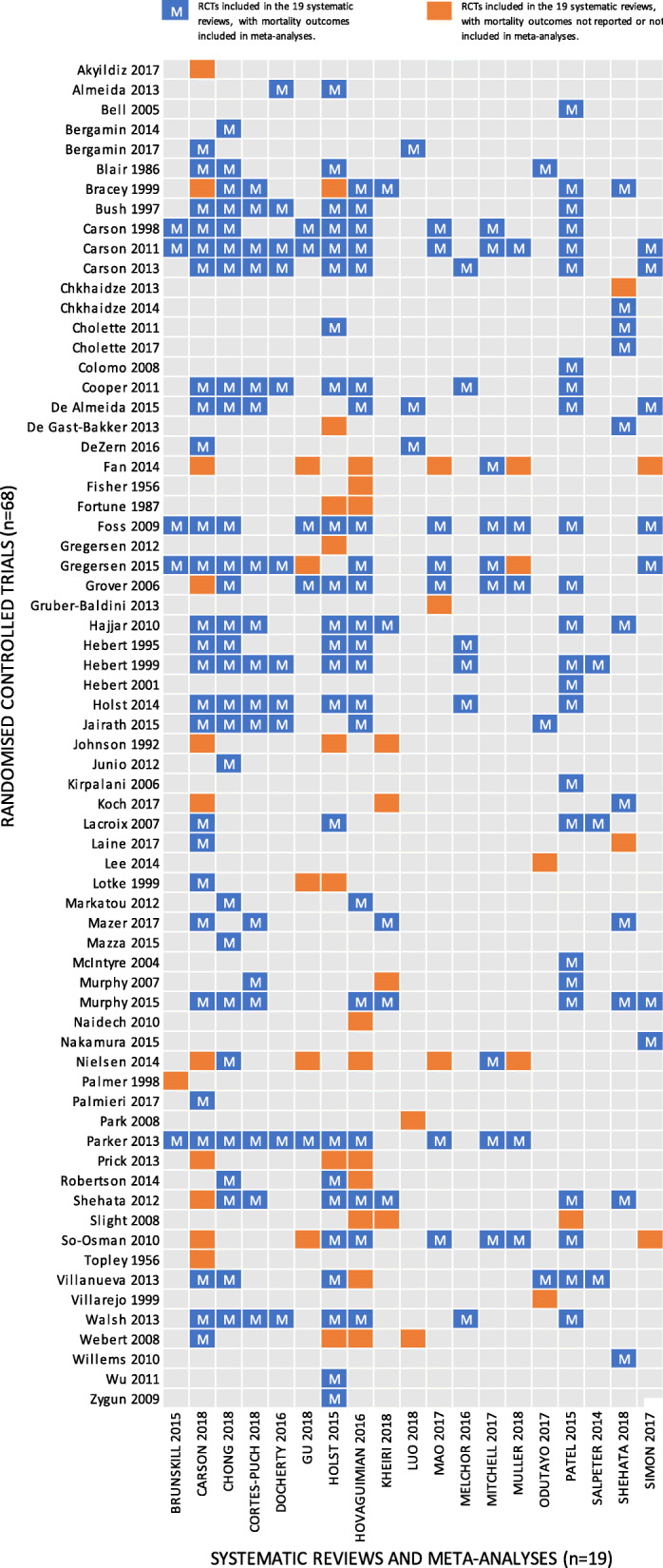
Matrix of randomised controlled trials pooled by the 19 included systematic reviews and meta-analyses

Figure 2 presents a matrix of unique RCTs pooled by the 19 systematic reviews and meta-analyses. Significant overlap between the included systematic reviews and meta-analyses exists. Sixty-eight unique RCTs were pooled by the included systematic reviews and meta-analyses. Of these 68, 53 unique RCTs were included in the pooled analyses for mortality. Unique RCTs were included in a median of two meta-analyses (range one to 13).

### Comparison of patients transfused

Eight of the 19 reviews reported the pooled proportion of patients transfused between restrictive and liberal groups [8, 40, 46, 48–50, 53, 55], with a further three reviews providing enough information for the difference to be calculated [39, 43, 56]. In the restrictive arm, the proportion of patients transfused ranged from 36% [48, 50] to 63% [53], while in the liberal arm the proportion ranged from 74% [48] to 99% [53].

### Units of blood transfused

Seven of the 19 reviews reported the pooled mean difference in units of red cells transfused between restrictive and liberal transfusion arms [8, 41, 47, 49, 51, 54, 55], with the mean difference ranging from 0.33 [47] to 1.98 [54] mean units. The smallest difference in mean units transfused was from the meta-analysis by Luo et al. [﻿47﻿]. The largest difference in mean units was from the systematic review by Salpeter et al. [54]. Among the individual RCTs pooled, the smallest mean difference in units of red cells transfused between groups was 0.08, comparing a mean of 0.78 units transfused in the restrictive arm to a mean of 0.86 units transfused in the liberal [57]. These differences are largely explained by the definition of restrictive and liberal haemoglobin thresholds used in the included studies.

### Haemoglobin concentration

**Fig. 3:**
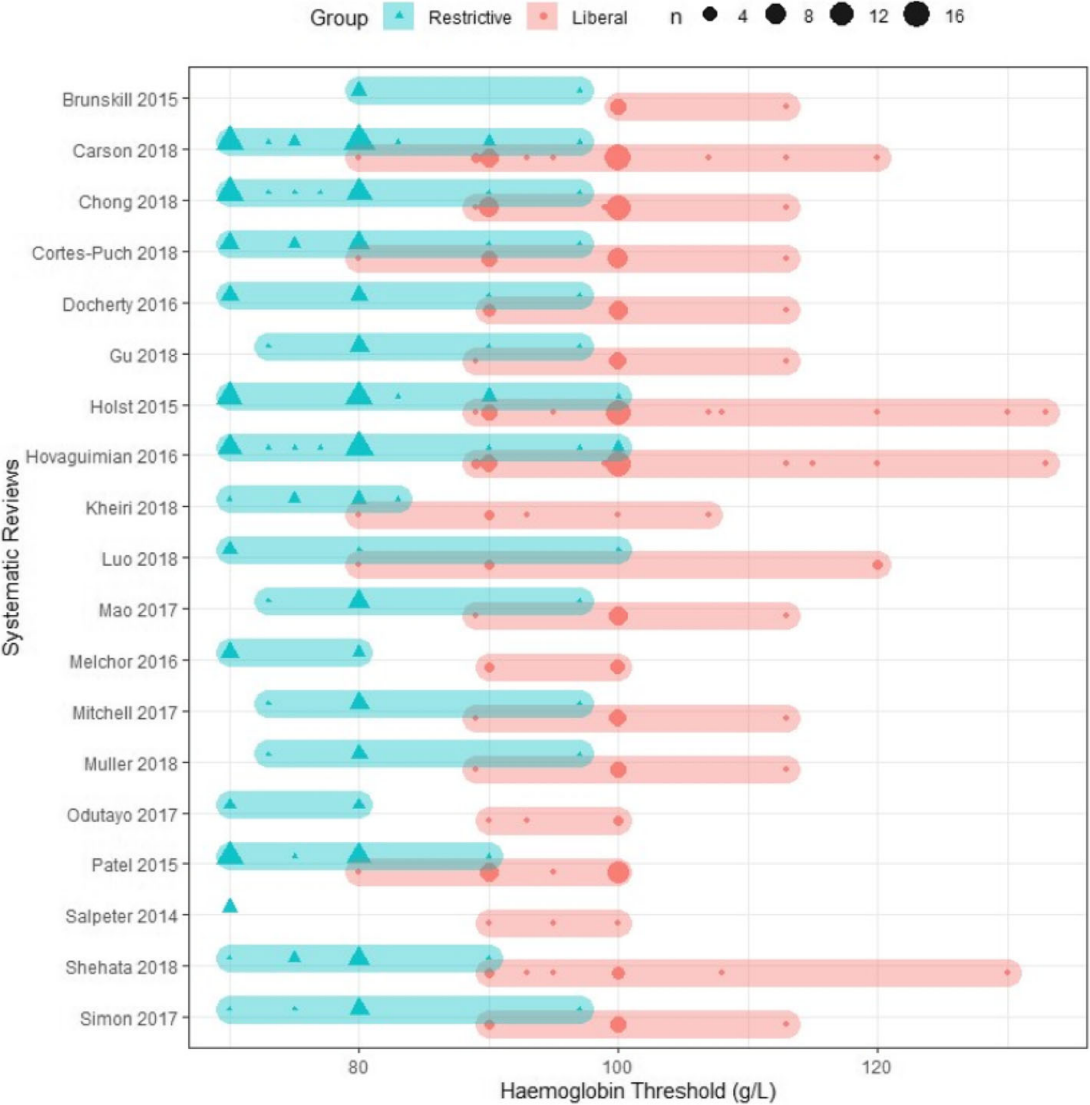
Range in restrictive and liberal haemoglobin thresholds pooled by systematic reviews and meta-analyses. Size of the markers represents number of trials

Almost all reviews reported the pooled difference in *planned* intervention haemoglobin thresholds for restrictive and liberal groups. This difference ranged from 0 to 30 g/L among restrictive groups, with one study [54] only pooling trials with restrictive thresholds below 70 g/L and three studies [8, 45, 47] pooling trials with restrictive thresholds ranging from 70 to 100 g/L. The difference in thresholds in the liberal groups ranged from 10 to 53 g/L, with four studies pooling trials with liberal thresholds ranging from 90 to 100 g/L [51–54] and one study pooling trials ranging from 80 to 133 g/L (Fig. 3) [45].

Only one review described the *actual* difference in mean haemoglobin thresholds between groups [39]. This review reported the result from one trial with a *planned* difference in haemoglobin thresholds of 20 g/L, with a threshold of below 80 g/L for the restrictive group and below 100 g/L for liberal. The *actual* mean difference in haemoglobin level before transfusion in this trial was smaller, with a 13 g/L higher mean difference in the liberal group.

### Transfusion dosing regimens

There was a lack of clarity around transfusion dosing regimens pooled between trials. Three reviews described the different planned post-transfusion haemoglobin targets or planned units of blood to be transfused, according to the trial protocols [39, 40, 53]. The criteria for and amount transfused differed between trials and within trials.

### Timing of randomisation

The timing of randomisation varied between individual trials, with some interventions commencing on admission, some intraoperatively, some after surgery, and others during a portion of a patient’s hospital stay; however, this information was reported only in four reviews [39, 46, 53, 55].

### Assessment of methodological quality and quality of evidence

**Fig. 4:**
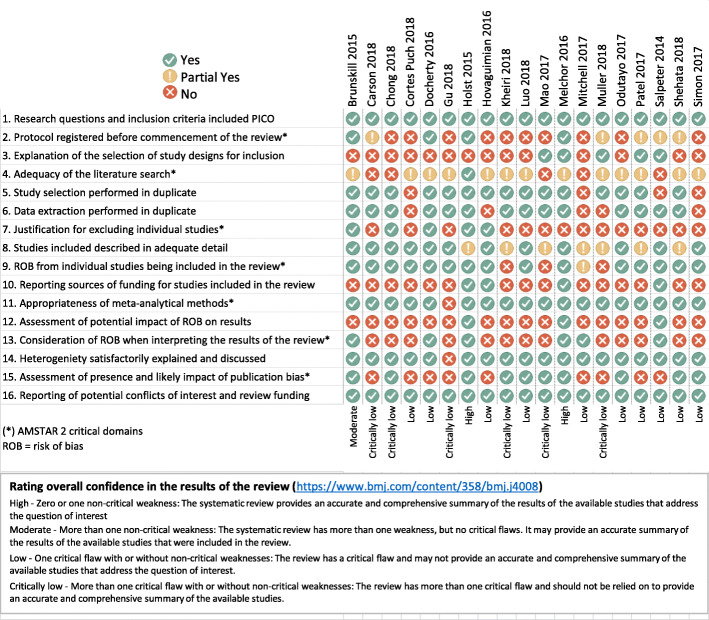
Assessing methodological quality of systematic reviews

**Fig. 5:**
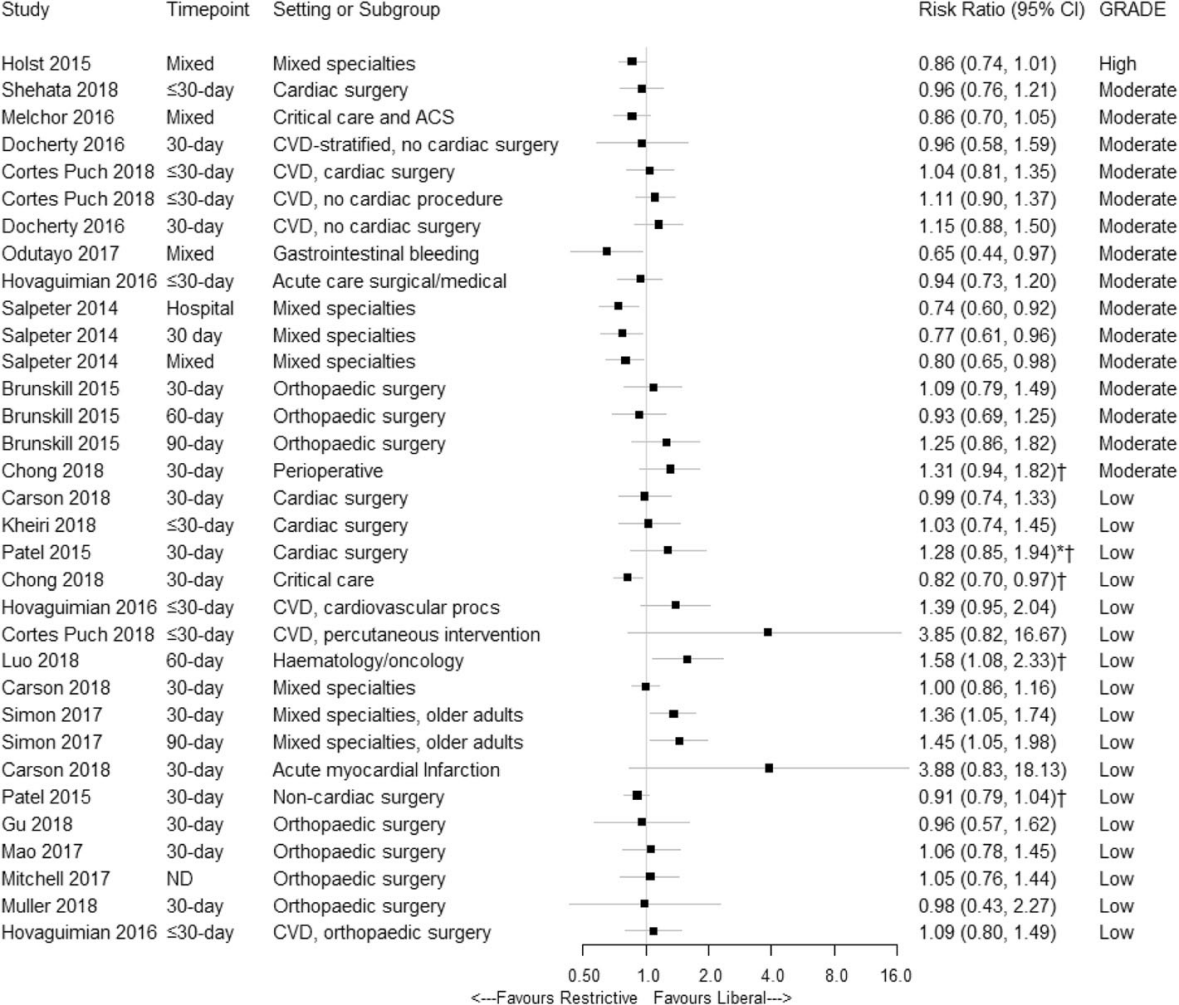
Relative risks (95% CI) for mortality from meta-analyses comparing restrictive and liberal red cell transfusion thresholds. ND = not defined; ^†^, odds ratios presented; *, odds ratio recalculated to reflect 30-day mortality

Detailed information on the methodological quality of included systematic reviews and meta-analyses is provided in Fig. 4. Using the 16 domains of the AMSTAR 2 tool, two reviews were of a high quality [8, 53], one was moderate [39], 10 were low [42, 43, 45, 47, 49, 51, 52, 54–56], and six were critically low [40, 41, 44, 46, 48, 50]. Figure 5 presents the assessment of the quality of evidence using the GRADE tool. The 19 included systematic reviews presented 33 meta-analyses on mortality. Of the 33 meta-analyses, one was high quality, 15 were moderate, 17 were low, and none were very low. More detailed information is available in Additional file 1: Table S5. The reasons for downgrading the quality of evidence to moderate or low included poor methodological quality as measured by AMSTAR 2, heterogeneity between study results, the inclusion of trials with a high risk of bias, and low numbers of participants pooled.

### Mortality

The most common mortality time point pooled was 30 days with 11 reviews presenting a meta-analysis of 30-day mortality [39–41, 43, 44, 46, 48, 50, 52, 54, 56] and a further three reviews pooling mortality within 30 days [42, 45, 55]. Four reviews presented a meta-analysis of mixed mortality time points [8, 51, 53, 54], two reviews presented the results of 60-day mortality [39, 47], two reviews of 90-day mortality [39, 56], one review captured in-hospital mortality [54], and one review did not describe the mortality time points pooled [49].

We contacted one study author to clarify the mortality time point pooled in their meta-analysis of cardiac surgery. The main outcome for this systematic review was 30-day mortality; however, the study applied the 90-day mortality from one large trial [52]. As this trial was likely to significantly influence the pooled estimate, we present a re-calculated odds ratio for 30-day mortality (Additional file 1: Table S6).

Figure 5 presents the relative risks for mortality in patients assigned to restrictive transfusion thresholds when compared with liberal thresholds. Of the 33 meta-analyses, 25 (75.8%) reported no statistically significant difference between restrictive and liberal transfusion thresholds, with significance set at the 5% level. Five (15.1%) reported restrictive transfusion thresholds resulted in significantly fewer deaths, while 3 (9.1%) reported liberal transfusion strategies resulted in fewer deaths.

### High to moderate GRADE ratings

Of the 33 meta-analyses identified, 16 were graded as high- to moderate-quality of evidence. Of these, 12 (75.0%) reported no statistically significant difference in mortality between restrictive and liberal transfusion groups and four (25.0%) reported significantly lower mortality for patients assigned to a restrictive transfusion strategy.

Only the meta-analysis by Holst et al. [8] was graded as high-quality evidence for mortality. This review reported a risk ratio for mortality of 0.86 with a restrictive transfusion strategy (95% CI 0.74 to 1.01, *p* = 0.07) when compared with a liberal strategy. The four meta-analyses graded as moderate quality of evidence, reporting significantly fewer deaths in patients assigned to a restrictive transfusion strategy were from two systematic reviews [51, 54]. One of these reviews [51] only included patients with gastrointestinal bleeding. The other meta-analyses were in the review by Salpeter et al. [54]. This systematic review differed from all the other reviews as it included trials where the restrictive strategy only allowed red cell transfusion at haemoglobin concentration of less than 70 g/L.

## Discussion

Our overview identified 19 systematic reviews reporting 33 meta-analyses comparing mortality in patients assigned to restrictive and liberal transfusion strategies. Among meta-analyses with high- to moderate-quality evidence for mortality, 75% reported no statistically significant difference between patients assigned to restrictive and liberal transfusion strategies, while 25% reported lower mortality with restrictive transfusion strategies. The meta-analyses reporting significantly fewer deaths in patients assigned to restrictive transfusion strategies differed in that one only included patients with gastrointestinal bleeding, while the others only evaluated patients assigned to a more restrictive haemoglobin threshold (below 70 g/L).

The identified meta-analyses with high- to moderate-quality evidence for mortality studied the following clinical settings: cardiac surgery, non-cardiac surgery, cardiovascular disease with and without cardiac surgery, critical care, gastrointestinal bleeding, and mixed clinical settings. Meta-analyses pooling mixed clinical settings included similar patient groups in addition to septic shock, trauma, and acute myocardial infarction. Although there may be limits to generalisability in some specific patient populations, the results from these studies indicate that a restrictive compared to a liberal transfusion strategy in most patient populations reduces the number of patients exposed to red cell transfusion and the number of red cell units transfused, and show that no difference in mortality exists.

These findings support the conclusions of evidence-based guidelines and reassure clinicians that restrictive transfusion thresholds can be applied in most patient populations. For example, guidelines from Australia, based on systematic reviews of available evidence, conclude that transfusion is usually inappropriate at haemoglobin concentrations above 90 g/L, and between 70 and 90 g/L transfusion is not associated with reduced mortality. Below 70 g/L transfusion may be appropriate; however, it may not be required in well-compensated patients where other specific therapy is available [58–62]. A number of other clinical practice guidelines and reviews recommend restrictive thresholds of 70 to 80 g/L for most clinical settings [63–65]. The results of future high-quality research may address uncertainty in specific clinical settings such as patients with acute coronary syndrome and haematology/oncology patients.

### Limitations of systematic reviews and meta-analyses

The quality of RCTs pooled by systematic reviews and meta-analyses are generally appraised through tools designed to assess the risk of bias and the quality of evidence. These tools focus on important elements in the conduct of RCTs such as concealed allocation, blinding, random allocation, selective reporting, statistical inconsistency (or heterogeneity), imprecision, indirectness, and publication bias.

While these domains are important in the general context of reviewing RCTs, we identified key clinical differences in the RCTs comparing transfusion strategies. Some, including variability in the study setting, variability in the patient population, and variability in the timing of the outcome measure, apply to all systematic reviews. Four, however, are unique to this topic: (1) haemoglobin thresholds selected for transfusion, (2) the absolute difference of pre-transfusion haemoglobin concentrations, (3) time to randomisation (resulting in transfusion administered prior to randomisation), and (4) comparable transfusion dosing regimens.

We identified examples of systematic reviews attempting to address aspects of these issues. For example, the systematic review by Salpeter et al. [54] addressed differences in restrictive haemoglobin thresholds pooled by including trials with the same threshold for restrictive transfusions. This may explain why their finding of significantly lower mortality differed to the majority of other meta-analyses. However, the majority of systematic reviews pooled trials with a variety of restrictive and liberal transfusion strategies that included haemoglobin concentrations ranging from 70 to 100 g/L and 80 to 133 g/L. This means the haemoglobin concentrations used in some restrictive strategies were higher than those in some liberal strategies.

None of the systematic reviews and meta-analyses included in this overview accounted for the variation in absolute difference in haemoglobin threshold concentrations between restrictive and liberal protocols within trials. This varied considerably with systematic reviews pooling trials with a difference from 10 g/L (including trials comparing restrictive thresholds of 70, 80, or 90 g/L with liberal thresholds of 80, 90, or 100 g/L respectively) to 40 g/L (including a trial comparing a restrictive threshold of 80 g/L with 120 g/L). Further confounding this issue is the change in difference between haemoglobin thresholds defined in transfusion protocols and the *actual* resulting difference in haemoglobin concentration prior to transfusion observed throughout the trial. For example, one systematic review [39] reported that one trial compared a difference in haemoglobin concentration thresholds between transfusion protocols of 20 g/L (80 g/L compared with 100 g/L); however, the actual mean difference in haemoglobin level before transfusion was 13 g/L.

One systematic review [﻿55﻿] addressed the important issue of timing of randomisation in their review of cardiac surgery RCTs by conducting a sub-group analysis of trials randomised intraoperatively and trials randomised postoperatively. In some trials, many participants have had a red cell transfusion prior to randomisation that is not included in the analysis. This potentially introduces bias into the estimate of risk for the specified outcome.

The least discussed of the identified issues was the lack of comparable transfusion dosing regimens between studies. Some RCTs set protocols for post transfusion haemoglobin targets to determine the amount of blood to transfuse, others indicated the number of units to transfuse when the haemoglobin threshold was met, and others were unclear. Of the 19 systematic reviews we included, only three referred to these differences. These differences may explain the large variation in the actual number of units transfused between restrictive and liberal groups between studies pooled, which was as low as a mean difference of less than half a unit in one systematic review to a high of two units in another.

These issues are relevant because they may result in smaller than expected differences in transfusion rates and transfused units between groups, and skew the results towards no statistically significant differences in outcome. We recommend that systematic reviews and meta-analyses comparing outcomes in restrictive and liberal transfusion trials be interpreted with these issues in mind.

### Limitations of our overview

A potential limitation of our overview of systematic reviews was that we restricted our literature search to reviews published in the English language. It is unlikely this has had any substantive influence on our findings as 16 of the 19 included systematic reviews had an unrestricted search by language.

We limited our search to systematic reviews and meta-analyses published between 2008 and 2018. We made this decision because systematic reviews and meta-analyses are frequently updated as new trials are published. However, this restriction did not mean trials published prior to 2008 were excluded, as the systematic reviews and meta-analyses included in our overview pooled trials without date restrictions.

We restricted the primary outcome of our overview to mortality, and as a result, any morbidity outcomes reported were not included. Though these outcomes are important, and frequently reported in trials as secondary outcomes, they have limitations. Authors of systematic reviews have highlighted this limitation and recommended caution in interpretation due to their subjective nature [8–10].

## Conclusions

This overview of reviews evaluating the effect on mortality of restrictive and liberal transfusion strategies identified 19 systematic reviews and 33 meta-analyses that used data from 53 RCTs. Of the 33 meta-analyses, one was graded as high quality, 15 were moderate, and 17 were low. The 16 meta-analyses of mortality graded as high to moderate quality demonstrate no difference in mortality between these two transfusion strategies.

Australian guidelines state that red cell transfusion should not be dictated solely by haemoglobin concentration; rather, the decision should also be based on a patient’s clinical signs and symptoms, the availability of other therapies for the treatment of anaemia, and the presence of risk factors for haemorrhage [58–62]. Similarly, the AABB Clinical Practice Guidelines highlight it is a good practice to individualise transfusion decisions taking into account the overall clinical context, haemoglobin decline, symptoms, volume status, patient preferences, and alternative therapies [63]. Therefore, important questions for future research include examining whether haemoglobin concentration is a good indicator of tissue oxygen needs and looking at other physiological parameters to assess whether these would be more indicative of tissue oxygenation and perfusion.

Should future systematic reviews and meta-analyses comparing transfusion thresholds be updated, we recommend they address the variations between studies in haemoglobin thresholds selected for transfusion, the gap in haemoglobin thresholds between restrictive and liberal groups both planned and actual, the different timing of randomisation, and the lack of comparable transfusion dosing regimens.

## Supplementary information

**Additional file 1.** Medline search strategy. **Table S1.** AMSTAR 2 form. **Table S2.** Algorithm developed to assign GRADE levels of evidence for overviews. **Table S3.** Grade levels definition. **Table S4.** Excluded reviews with exclusion reasons. **Table S5.** Results from algorithm developed to assign GRADE levels of evidence for overviews. **Table S6.** Recalculated odds ratio for 30-day mortality from Patel et al. meta-analysis.

## Data Availability

All data generated or analysed during this study are included in this published article and supplementary material.
