# Supplementary material for: Systematic reviews and meta-analyses comparing mortality in restrictive and liberal haemoglobin thresholds for red cell transfusion: an overview of systematic reviews
Source: BMC Med. 2020 Jun 24;18:154. doi: 10.1186/s12916-020-01614-w (PMC7313211; doi:10.1186/s12916-020-01614-w)
Supplement: Supplementary file 1 — Additional file 1. Medline search strategy. Table S1. AMSTAR 2 form. Table S2. Algorithm developed to assign GRADE levels of evidence for overviews. Table S3. Grade levels definition. Table S4. Excluded reviews with exclusion reasons. Table S5. Results from algorithm developed to assign GRADE levels of evidence for overviews. Table S6. Recalculated odds ratio for 30-day mortality from Patel et al. meta-analysis. [file 12916_2020_1614_MOESM1_ESM.docx]

**Additional file 1**

Supplement to: Overview of systematic reviews and meta-analyses comparing mortality in restrictive and liberal haemoglobin thresholds for red cell transfusion.

**Medline Search Strategy**

**Ovid MEDLINE(R) 1946 to Present with Daily Update (MESD)**

1 *Blood transfusion/

2 *Erythrocyte Transfusion/

3 1 or 2

4 (threshold* or targeted or trigger* or restrict* or liberal* or aggressive or conservativ*).mp.

5 3 and 4

6 transfus*.mp.

7 ((red cell* or red blood cell* or RBC* or PRBC* or h?emoglobin or h?emocrit or HB or HCT) adj3 (threshold* or

targeted or trigger* or restrict* or liberal* or aggressiv* or conservativ*)).mp.

8 6 and 7

9 5 or 8

10 exp Mortality/

11 Treatment outcome/

12 mo.fs.

13 (mortality or outcome*).mp.

14 10 or 11 or 12 or 13

15 9 and 14

16 "systematic review"/

17 15 and 16

18 ((("systematic review" or "systematic reviews" or metaanaly* or meta-analy* or systematic literature review* or

meta synthesis or metasynthesis or overview* or (cochrane adj2 review*) or (umbrella adj2 review*) or unpublished or

citation* or references or scales or papers or datasets or rapid review or evidence synthesis or (integrative adj2 review*)).mp. or consensus development conference.pt.) and ((literature or articles or publications or publication or bibliography or published or database* or trials or internet or textbooks or (clinical and studies)).ab,ti. or treatment

outcome*.mp.)) not (letter or newspaper article).pt.

19 15 and 18

20 limit 15 to (meta analysis or systematic reviews)

21 17 or 19 or 20

22 limit 21 to english language

23 limit 22 to yr="2008 -Current"

**Additional file 1: Table S1. AMSTAR 2 form**(Shea BJ, Reeves BC, Wells G, Thuku M, Hamel C, Moran J, Moher D, Tugwell P, Welch V, Kristjansson E, Henry DA. AMSTAR 2: a critical appraisal tool for systematic reviews that include randomised or non-randomised studies of healthcare interventions, or both. BMJ. 2017 Sep 21;358:j4008.)

| AMSTAR 2 | | | |
| --- | --- | --- | --- |
| **1. Did the research questions and inclusion criteria for the review include the components of PICO?** | | | |
| For Yes: | | Optional (recommended) | |
| 🞎 | Population | 🞎 | Timeframe for follow up |
| 🞎 | Intervention |  |  |
| 🞎 | Comparator group |  |  |
| 🞎 | Outcome |  |  |
| **2. Did the report of the review contain an explicit statement that the review methods were established prior to the conduct of the review and did the report justify any significant deviations from the protocol?** | | | |
| For Partial Yes: The authors state that they had a written protocol or guide that included ALL the following: | | For Yes: As for partial yes, plus the protocol should be registered and should also have specified: | |
| 🞎 | review question(s) | 🞎 | a meta-analysis/synthesis plan, if appropriate, and |
| 🞎 | a search strategy | 🞎 | a plan for investigating causes of heterogeneity |
| 🞎 | inclusion/exclusion criteria |  | justification for any deviations from the protocol |
| 🞎 | a risk of bias assessment |  |  |
| **3. Did the review authors explain their selection of the study designs for inclusion in the review?** | | | |
| For Yes, the review should satisfy ONE of the following: | | | |
| 🞎 | Explanation for including only RCTs | | |
| 🞎 | OR Explanation for including only NRSI | | |
| 🞎 | OR Explanation for including both RCTs and NRSI | | |
| **4. Did the review authors use a comprehensive literature search strategy?** | | | |
| For Partial Yes (all the following): | | For Yes, should also have (all the following): | |
| 🞎 | searched at least 2 databases (relevant to research question) | 🞎 | searched the reference lists / bibliographies of included studies |
| 🞎 | provided key word and/or search strategy | 🞎 | searched trial/study registries |
| 🞎 | justified publication restrictions (e.g. language) | 🞎 | included/consulted content experts in the field |
|  | | 🞎 | where relevant, searched for grey literature |
|  |  | 🞎 | conducted search within 24 months of completion of the review |
| **5. Did the review authors perform study selection in duplicate?** | | | |
| For Yes, either ONE of the following: | | | |
| 🞎 | at least two reviewers independently agreed on selection of eligible studies and achieved consensus on which studies to include | | |
| 🞎 | OR two reviewers selected a sample of eligible studies and achieved good agreement (at least 80 percent), with the remainder selected by one reviewer. | | |
| **6. Did the review authors perform data extraction in duplicate?** | | | |
| For Yes, either ONE of the following: | | | |
| 🞎 | at least two reviewers achieved consensus on which data to extract from included studies | | |
| 🞎 | OR two reviewers extracted data from a sample of eligible studies and achieved good agreement (at least 80 percent), with the remainder extracted by one reviewer. | | |
| **7. Did the review authors provide a list of excluded studies and justify the exclusions?** | | | |
| For Partial Yes: | | For Yes, must also have: | |
| 🞎 | provided a list of all potentially relevant studies that were read in full-text form but excluded from the review | 🞎 | Justified the exclusion from the review of each potentially relevant study |
| **8. Did the review authors describe the included studies in adequate detail?** | | | |
| For Partial Yes (ALL the following): | | For Yes, should also have ALL the following: | |
| 🞎 | described populations | 🞎 | described population in detail |
| 🞎 | described interventions | 🞎 | described intervention in detail (including doses where relevant) |
| 🞎 | described comparators | 🞎 | described comparator in detail (including doses where relevant) |
| 🞎 | described outcomes | 🞎 | described study’s setting |
| 🞎 | described research designs | 🞎 | timeframe for follow-up |
| **9. Did the review authors use a satisfactory technique for assessing the risk of bias (RoB) in individual studies that were included in the review?** | | | |
| For Partial Yes, must have assessed RoB from | | For Yes, must also have assessed RoB from: | |
| 🞎 | unconcealed allocation, and | 🞎 | allocation sequence that was not truly random, and |
|  |  |  |  |
| 🞎 | lack of blinding of patients and assessors when assessing outcomes (unnecessary for objective outcomes such as all-cause mortality) | 🞎 | selection of the reported result from among multiple measurements or analyses of a specified outcome |
| **10. Did the review authors report on the sources of funding for the studies included in the review?** | | | |
| For Yes: | | | |
| 🞎 | Must have reported on the sources of funding for individual studies included in the review. Note: Reporting that the reviewers looked for this information but it was not reported by study authors also qualifies | | |
|  |  |  |  |
| **11. If meta-analysis was performed did the review authors use appropriate methods for statistical combination of results?** | | | |
| For Yes: | | | |
| 🞎 | The authors justified combining the data in a meta-analysis | | |
| 🞎 | AND they used an appropriate weighted technique to combine study results and adjusted for heterogeneity if present. | | |
| 🞎 | AND investigated the causes of any heterogeneity | | |
| **12. If meta-analysis was performed, did the review authors assess the potential impact of RoB in individual studies on the results of the meta-analysis or other evidence synthesis?** | | | |
| For Yes: | | | |
| 🞎 | included only low risk of bias RCTs | | |
| 🞎 | OR, if the pooled estimate was based on RCTs at variable RoB, the authors performed analyses to investigate possible impact of RoB on summary estimates of effect. | | |
| **13. Did the review authors account for RoB in individual studies when interpreting/ discussing the results of the review?** | | | |
| For Yes: | | | |
| 🞎 | included only low risk of bias RCTs | | |
| 🞎 | OR, if RCTs with moderate or high RoB were included, the review provided a discussion of the likely impact of RoB on the results | | |
| **14. Did the review authors provide a satisfactory explanation for, and discussion of, any heterogeneity observed in the results of the review?** | | | |
| For Yes: | | | |
| 🞎 | There was no significant heterogeneity in the results | | |
| 🞎 | OR if heterogeneity was present the authors performed an investigation of sources of any heterogeneity in the results and discussed the impact of this on the results of the review | | |
| **15. If they performed quantitative synthesis did the review authors carry out an adequate investigation of publication bias (small study bias) and discuss its likely impact on the results of the review?** | | | |
| For Yes: | | | |
| 🞎 | performed graphical or statistical tests for publication bias and discussed the likelihood and magnitude of impact of publication bias | | |
| **16. Did the review authors report any potential sources of conflict of interest, including any funding they received for conducting the review?** | | | |
| For Yes: | | | |
| 🞎 | The authors reported no competing interests OR | | |
| 🞎 | The authors described their funding sources and how they managed potential conflicts of interest | | |

**Additional file 1: Table S2. Algorithm specifically developed to assign GRADE levels of evidence for overviews.**(From Pollock A, Farmer SE, Brady MC, Langhorne P, Mead GE, Mehrholz J, et al. An algorithm was developed to assign GRADE levels of evidence to comparisons within systematic reviews. J Clin Epidemiol. 2016;70:106-10.)

|  |  |  | |  |  | |
| --- | --- | --- | --- | --- | --- | --- |
| Area assessed | Imprecision | Risk of bias  (trial quality) | | Inconsistency | Risk of bias  (review quality) | |
| Method of assessment | Number of participants within pooled analysis | Proportion of participants included in the pooled analysis judged to have low ROB for randomization and observer blinding | | Heterogeneity,  assessed by *I^2^* statistic | Responses to AMSTAR questions 1-4 (covering a priori research design, search characteristics, independence of study selection and data extraction) | |
| No downgrade  (no serious limitations) | ≥ 200 | ≥ 75% of participants have low ROB | | *I^2^* ≤ 75% | 4/4 are all "yes" (i.e., low ROB) | |
| Downgrade 1 level  (serious limitations) | 100-199 | < 75% of participants have low ROB | | *I^2^* > 75% | 3/4 are "yes" and 1 is "unclear" or "no" on AMSTAR | |
| Downgrade 2 levels  (very serious limitations) | 1-99 |  | |  | < 3/4 are "yes" and remainder are "unclear" or "no" on AMSTAR | |
| Notes |  | If ROB for individual trials was not reported within the review, we were conservative and assumed that less than 75% of participants had low ROB. | | If only one trial contributed to analysis, no downgrade; if *I^2^* not reported, assumed to be greater than 75%. |  | |
| Formula for applying GRADE level of evidence from number of downgrades determined using the algorithm | | | | | |  |
| GRADE level of evidence | | | Number of downgrades (derived from objective assessment) | | | |
| High | | | 0 downgrade | | | |
| Moderate | | | 1 or 2 downgrades | | | |
| Low | | | 3 or 4 downgrades | | | |
| Very low | | | 5 or 6 downgrades | | | |

**Additional file 1: Table S3. Grade levels definition**(From Table 5.1, Schünemann H, Brożek J, Guyatt G, Oxman A. GRADE Handbook: Handbook for grading the quality of evidence and the strength of recommendations using the GRADE approach. 2013) <https://gdt.gradepro.org/app/handbook/handbook.html>.

|  |  |
| --- | --- |
| Grade | Definition |
| High | We are very confident that the true effect lies close to that of the estimate of the effect. |
| Moderate | We are moderately confident in the effect estimate: The true effect is likely to be close to the estimate of the effect, but there is a possibility that it is substantially different. |
| Low | Our confidence in the effect estimate is limited: The true effect may be substantially different from the estimate of the effect. |
| Very Low | We have very little confidence in the effect estimate: The true effect is likely to be substantially different from the estimate of effect. |

**Additional file 1: Table S4. Excluded reviews with exclusion reasons.**

| First Author | Year | Journal | Exclusion Reason |
| --- | --- | --- | --- |
| Carson | 2016 | Cochrane Database of Systematic Reviews | More recent review published |
| Curley | 2014 | Critical Care Medicine | More recent review published |
| Puch | 2017 | American Journal of Respiratory and Critical Care Medicine | Abstract |
| Dupuis | 2017 | Annals of Intensive Care | Mortality data from one trial |
| Fominskiy | 2015 | British Journal of Anaesthesia | More recent review published |
| Hoeks | 2017 | British Journal of Haematology | Mortality data from one trial |
| Desjardins | 2012 | Canadian Journal of Anesthesia | Abstract |
| Ahn | 2016 | Canadian Journal of Emergency Medicine | Abstract |
| Prescott | 2016 | Cancer Treatment Reviews | Pooled non-randomized trials |
| Chatterjee | 2012 | Circulation | Abstract |
| Carson | 2012 | Cochrane Database of Systematic Reviews | More recent review published |
| Wilkinson | 2014 | Cochrane Database of Systematic Reviews | Mortality data from one trial |
| Gu | 2015 | Cochrane Database of Systematic Reviews | Mortality data from one trial |
| Estcourt | 2017 | Cochrane Database of Systematic Reviews | Mortality data from one trial |
| Carless | 2010 | Cochrane Database of Systematic Reviews | More recent review published |
| Desjardins | 2012 | Critical Care | Not a meta-analysis |
| Docherty | 2016 | Critical Care | Abstract |
| Wang | 2017 | Current Medical Research & Opinion | Pooled non-randomized trials |
| Carson | 2013 | JAMA | Not a meta-analysis |
| Chatterjee | 2013 | JAMA Internal Medicine | Pooled non-randomized trials |
| Gu | 2015 | Leukemia Research | Abstract |
| Desjardins | 2011 | Neurocritical Care | Abstract |
| Holst | 2014 | New England Journal of Medicine | Not a meta-analysis |
| Wang | 2013 | World Journal of Gastroenterology | More recent review published |

**Additional file 1: Table S5. Results from algorithm developed to assign GRADE levels of evidence for overviews.**(From Pollock A, Farmer SE, Brady MC, Langhorne P, Mead GE, Mehrholz J, et al. An algorithm was developed to assign GRADE levels of evidence to comparisons within systematic reviews. J Clin Epidemiol. 2016;70:106-10.)

|  | Mortality Pooled | Number of participants | % of participants in pooled analysis with low ROB for randomisation | I² | No. of "Yes" Responses to AMSTAR Qs 2,4,5,6 | No. of Downgrades | GRADE level of evidence |
| --- | --- | --- | --- | --- | --- | --- | --- |
| Brunskill 2015 | 30-day, Orthopaedic surgery | 2683 | 3 | 38 | 3 | -2 | Moderate |
| Brunskill 2015 | 60-day, Orthopaedic surgery | 2283 | 4 | 0 | 3 | -2 | Moderate |
| Brunskill 2015 | 90-day, Orthopaedic surgery | 484 | 0 | 0 | 3 | -2 | Moderate |
| Carson 2018 | 30-day, Mixed specialties | 15681 | 39 | 33 | 2 | -3 | Low |
| Carson 2018 | 30-day, Cardiac surgery | 7441 | 7 | 14 | 2 | -3 | Low |
| Carson 2018 | 30-day, Acute myocardial infarction | 154 | 71 | 0 | 2 | -4 | Low |
| Chong 2018 | 30-day, Critical care | 4332 | 70 | 0 | 2 | -3 | Low |
| Chong 2018 | 30-day, Perioperative | 6410 | 81 | 20 | 2 | -2 | Moderate |
| Cortes Puch 2018 | ≤30-day, CVD no cardiac procedure | 2048 | 82 | 2 | 0 | -2 | Moderate |
| Cortes Puch 2018 | ≤30-day, CVD orthopaedic surgery | 154 | 71 | 0 | 0 | -4 | Low |
| Cortes Puch 2018 | ≤30-day, CVD cardiac surgery | 7343 | 90 | 0 | 0 | -2 | Moderate |
| Docherty 2016 | 30-day, CVD no cardiac surgery | 3303 | 68 | 14 | 3 | -2 | Moderate |
| Docherty 2016 | 30-day, CVD-stratified no cardiac surgery | 2289 | 92 | 14 | 3 | -1 | Moderate |
| Gu 2018 | 30-day, Orthopaedic surgery | 2680 | 0 | 76 | 2 | -4 | Low |
| Holst 2015 | Mixed time point, Mixed specialties | 5707 | 100 | 27 | 4 | 0 | High |
| Hovaguimian 2016 | ≤30-day, CVD cardiovascular procs | 3245 | 2 | 0 | 1 | -3 | Low |
| Hovaguimian 2016 | ≤30-day, CVD orthopaedic surgery | 3546 | 17 | 17 | 1 | -3 | Low |
| Hovaguimian 2016 | ≤30-day, Acute care surgical/medical | 2894 | 77 | 45 | 1 | -2 | Moderate |
| Kheiri 2018 | ≤30-day, Cardiac surgery | 7848 | 0 | 21 | 2 | -3 | Low |
| Luo 2018 | 60-day, Haematology/Oncology | 587 | 0 | 38 | 2 | -3 | Low |
| Mao 2017 | 30-day, Orthopaedic surgery | 3525 | 0 | 18 | 2 | -3 | Low |
| Melchor 2016 | Mixed time point, Critical care and ACS | 2159 | 0 | 41 | 3 | -2 | Moderate |
| Mitchell 2017 | ND, Orthopaedic surgery | 3760 | 71 | 28 | 0 | -3 | Low |
| Muller 2018 | 30-day, Orthopaedic surgery | 3157 | 0 | 29 | 1 | -3 | Low |
| Odutayo 2017 | Mixed time point, Gastrointestinal bleeding | 1578 | 97 | 0 | 2 | -2 | Moderate |
| Patel 2015 | 30-day, Cardiac surgery | 3304 | 62 | 0 | 2 | -3 | Low |
| Patel 2015 | 30-day, Non-cardiac surgery | 8341 | 53 | 30 | 2 | -3 | Low |
| Salpeter 2014 | Hospital Mortality, Mixed specialties | 1727 | 100 | 75 | 1 | -2 | Moderate |
| Salpeter 2014 | 30-day, Mixed specialties | 2364 | 100 | 75 | 1 | -2 | Moderate |
| Salpeter 2014 | Mixed time point, Mixed specialties | 2364 | 100 | 75 | 1 | -2 | Moderate |
| Shehata 2018 | ≤30-day, Cardiac surgery | 9092 | 77 | 0 | 2 | -2 | Moderate |
| Simon 2017 | 30-day, Mixed specialties older adults | 4969 | 43 | 59 | 0 | -3 | Low |
| Simon 2017 | 90-day, Mixed specialties older adults | 2287 | 0 | 0 | 0 | -3 | Low |

|  |  |  |  |  |  |  |  |  |  |  |
| --- | --- | --- | --- | --- | --- | --- | --- | --- | --- | --- |
| **Additional file 1: Table S6. Recalculated odds ratio for 30-day mortality from Patel et al. meta-analysis, using 30-day mortality from Murphy 2015** | | | | | | | | | | |
|  |  |  |  |  |  |  |  |  |  |  |
|  | Restrictive | | Liberal | |  |  |  |  |  |  |
| Study | Deaths | Total | Deaths | Total | Odds Ratio | 95% CI | z | P | Weight (%) | |
|  |  |  |  |  |  |  |  |  | Fixed | Random |
| Murphy 2015* | 26 | 1000 | 19 | 1003 | 1.382 | 0.760 to 2.514 |  |  | 49.13 | 49.13 |
| Shehata 2012 | 4 | 25 | 1 | 25 | 4.571 | 0.473 to 44.172 |  |  | 3.42 | 3.42 |
| Hajjar 2010 | 15 | 249 | 13 | 253 | 1.183 | 0.551 to 2.541 |  |  | 30.10 | 30.10 |
| Murphy 2007* | 5 | 162 | 3 | 159 | 1.656 | 0.389 to 7.049 |  |  | 8.38 | 8.38 |
| Bracey 1999 | 3 | 215 | 6 | 222 | 0.509 | 0.126 to 2.063 |  |  | 8.98 | 8.98 |
| Total (fixed effects) | 53 | 1651 | 42 | 1662 | 1.283 | 0.849 to 1.937 | 1.184 | 0.236 | 100.00 | 100.00 |
| Total (random effects) | 53 | 1651 | 42 | 1662 | 1.276 | 0.839 to 1.940 | 1.138 | 0.255 | 100.00 | 100.00 |
| *** Indicates figures have been updated** | | | | | | | | | | |
